# 5-Lipoxygenase (5-LO) is Involved in Kupffer Cell Survival. Possible Role of 5-LO Products in the Pathogenesis of Liver Fibrosis

**DOI:** 10.1186/1476-5926-2-S1-S19

**Published:** 2004-01-14

**Authors:** Esther Titos, Anna Planagumà, Marta Làpez-Parra, Neus Villamor, Rosa Miquel, Wladimiro Jimànez, Vicente Arroyo, Francisca Rivera, Joan Rodàs, Joan Clària

**Affiliations:** 1 DNA Unit, Hospital Clànic, Institut d'Investigacions Biomàdiques August Pi i Sunyer (IDIBAPS), Universitat de Barcelona, Barcelona 08036, Spain; 2 Hematopathology Laboratory, Hospital Clànic, Institut d'Investigacions Biomàdiques August Pi i Sunyer (IDIBAPS), Universitat de Barcelona, Barcelona 08036, Spain; 3 Pathology Laboratory, Hospital Clànic, Institut d'Investigacions Biomàdiques August Pi i Sunyer (IDIBAPS), Universitat de Barcelona, Barcelona 08036, Spain; 4 Hormonal Laboratory, Hospital Clànic, Institut d'Investigacions Biomàdiques August Pi i Sunyer (IDIBAPS), Universitat de Barcelona, Barcelona 08036, Spain; 5 Liver Unit, Hospital Clànic, Institut d'Investigacions Biomàdiques August Pi i Sunyer (IDIBAPS), Universitat de Barcelona, Barcelona 08036, Spain

## Introduction

A wealth of evidence indicates that inflammation plays a central role in the current paradigm of liver fibrosis. Kupffer cells, which represent the largest population of resident macrophages in the body [1], are uniquely positioned as the predominant primary inflammatory effector cells to initiate the inflammatory cascade leading to tissue remodeling and fibrosis. For this reason, the presence of an increased population of Kupffer cells together with the bulk release of inflammatory mediators by these macrophages are considered to be critical events during the early stages of liver inflammation and fibrosis [2,3].

Arachidonic acid metabolites derived from 5-lipoxygenase (5-LO) are essential regulators of cell growth and survival [4]. Given that we recently demonstrated that 5-LO expression and leukotriene (LT) formation are increased in livers from rats with carbon tetrachloride (CCl_4_)-induced cirrhosis [5], it is our hypothesis that 5-LO products play a role in Kupffer cell survival and in the pathogenesis of liver inflammation and fibrosis. Therefore, in the current study we examined the 5-LO pathway in sinusoidal liver cells and specifically analyzed the role of 5-LO in Kupffer cell growth and survival.

## Methods

### Experimental model of hepatic fibrosis

Liver injury was induced in male adult Wistar rats by inhalation of CCl_4 _as described elsewhere [6].

### Isolation and culture of Kupffer cells

Liver cells were isolated by *in situ *collagenase perfusion and purified by Percollà density gradients as previously described [5,7]. Kupffer cells were characterized by nonspecific esterase activity staining and by immunolabeling with the monoclonal antibody RPE-ED2 and cultured in RPMI 1640 supplemented with 2 mM L-glutamine, penicillin (50 U/ml), streptomycin (50 micrograms/ml) and 10% FCS [5].

### RNA isolation and RT-PCR

Total RNA was obtained by the guanidinium isothiocyanate-cesium chloride method. RT was performed using an avian myeloblastoma virus reverse transcriptase cDNA synthesis kit. PCR was performed using oligonucleotides designed from published rat 5-LO, 5-LO-activating protein (FLAP), LTC_4 _synthase and glyceraldehyde-3-phosphate-dehydrogenase (GAPDH) cDNA sequences. PCR products were analyzed by gel electrophoresis.

### Analysis of 5-LO products

LTB_4 _and LTC_4_/LTD_4_/LTE_4 _(cysteinyl-LT) levels were quantified in cell supernatans of freshly isolated rat Kupffer cells (1à2.8 à 10^6 ^cells) maintained in culture for 16 hours by specific EIA kits. 5-hydroxyeicosatetraenoic acid (5-HETE) was analyzed by RP-HPLC.

### Analysis of cell proliferation

Rat Kupffer cells (1à2.8 à 10^6 ^cells) were cultured for up to 6 days in complete RPMI 1640 medium and cell growth was determined by the microculture MTT assay. To ascertain the effects of 5-LO inhibitors on cell survival, Kupffer cells from cirrhotic livers were grown in the presence of vehicle, AA861 (10 micromolar) and BAY-X-1005 (30 micromolar) for 8 h at 37 degrees C and the number of cells examined by direct counting using the Neuebauer chamber. The effects of 5-LO inhibitors on cell proliferation were further assessed in THP-1 cells by the MTT assay.

### Apoptosis Assays

Nuclear morphology was assessed by optical microscopy visualization in Diff-Quik^à^-stained THP-1 cells exposed to vehicle, AA861 (10 micromolar) or BAY-X-1005 (30 micromolar) for 96 hours at 37 degrees C. DNA fragmentation was detected using the TACSà DNA Laddering kit and visualized by agarose gel electrophoresis.

### Analysis of DNA content by flow cytometry

THP-1 cells were incubated in the presence of vehicle, AA861 (10 micromolar) or BAY-X-1005 (30 micromolar) at 37 degrees C. After 72 h, cells were stained with propidium iodide and DNA content frequency cell cycle distribution analyzed by means of fluorescence-activated cell sorting (FACS) analysis.

## Results

**Figure 1:**
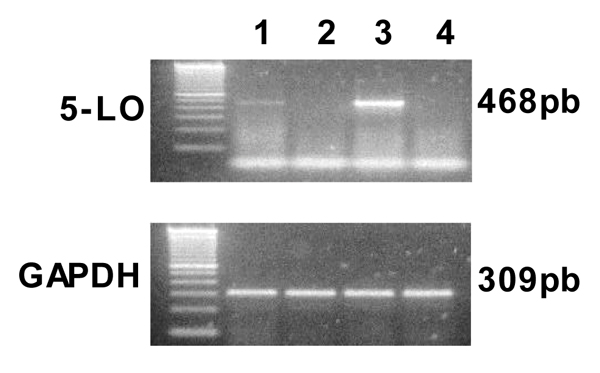
Representative RT-PCR analysis of 5-LO mRNA expression in rat liver cells. Lane 1, Kupffer cells; lane 2, hepatic stellate cells (HSC); lane 3, positive control; and lane 4, hepatocytes. GAPDH mRNA was used as housekeeping gene expression.

Among the different hepatic sinusoidal cell types, Kupffer cells have been historically considered to possess the capacity to produce most of liver arachidonic acid metabolites including the 5-LO products LTB_4 _and cysteinyl-LT [8]. Indeed, Kupffer cells are apparently the only liver sinusoidal cells endowed with the complete enzymatic set necessary for LT formation (see Figure 1 and Reference [5]).

Among the different 5-LO products, Kupffer cells generated significant amounts of LTB_4 _and cysteinyl-LT (Table 1). 5-HETE was not detected in these incubations. Interestingly, the ability to produce LTB_4 _was found to be increased in Kupffer cells from rats treated with CCl_4_.

**Table 1:** Generation of 5-LO-derived eicosanoids by Kupffer cells isolated from control and CCl_4_-treated rats. N.D., not detected. *, P &lt; 0.05 vs control.

	**Control**	**CCl_4_-treated**
LTB_4 _(pg/10^6 ^cells)	3.98 à 0.89	7.52 à 1.66*
LTC_4_/LTD_4_/LTE_4 _(pg/10^6 ^cells)	5.74 à 0.99	3.82 à 0.42
5-HETE (ng/10^6 ^cells)	N.D.	N.D.

These 5-LO derived products are essential for Kupffer cell survival, because the number of Kupffer cells in culture was significantly reduced by the selective 5-LO inhibitor AA861 (42.7 à 4.7 % inhibition) and by the FLAP inhibitor BAY-X-1005 (55.2 à 2.5% inhibition). These findings were further characterized in THP-1 cells where AA861 and BAY-X-1005 inhibited proliferation in a dose- and time-dependent fashion. In these cells, the antiproliferative effect was associated with induction of programmed cell death, as evaluated by using different techniques for apoptosis detection (see "Methods").

## Discussion

**Figure 2:**
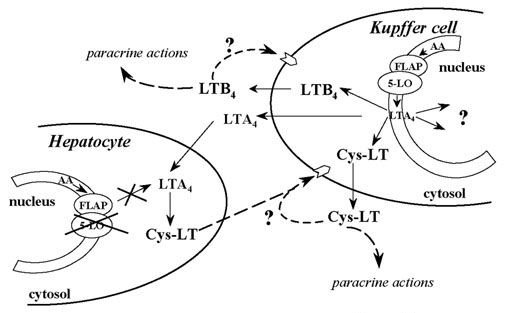
Biosynthesis of 5-LO products in sinusoidal liver cells

The current knowledge of the 5-LO pathway in liver sinusoidal cells is shown in figure 2. Kupffer cells, which constitutively express 5-LO, have the ability to produce LTB_4 _and cysteinyl-LT. Cysteinyl-LT are also produced in hepatocytes by transcellular metabolism of LTA_4 _formed by Kupffer cells [5]. Once synthesized, 5-LO-derived products may act in both paracrine and autocrine fashion modulating the contraction of nearby HSC or regulating macrophage cell growth. Interestingly, biosynthesis of 5-LO products is located in the nuclear cell membrane, where they can exert important nuclear functions. Taken together these data indicate that 5-LO plays an important role in cell proliferation and survival and set new ground for the application of 5-LO inhibitors during the inflammatory stage previous to the development of liver fibrosis.
